# Runoff Losses in Nitrogen and Phosphorus From Paddy and Maize Cropping Systems: A Field Study in Dongjiang Basin, South China

**DOI:** 10.3389/fpls.2021.675121

**Published:** 2021-08-10

**Authors:** Feifan Zeng, Zheng Zuo, Juncheng Mo, Chengyu Chen, Xingjian Yang, Jinjin Wang, Yi Wang, Zhongqiu Zhao, Tianyi Chen, Yongtao Li, Zhen Zhang, Zheng Hu, Huijuan Xu

**Affiliations:** ^1^College of Natural Resources and Environment, Joint Institute for Environmental Research and Education, South China Agricultural University, Guangzhou, China; ^2^Changsha Research Station for Agricultural and Environmental Monitoring and Key Laboratory of Agro-Ecological Processes in Subtropical Regions, Institute of Subtropical Agriculture, Chinese Academy of Sciences, Changsha, China; ^3^Ministry of Agriculture, Agro-Environmental Protection Institute, Tianjin, China

**Keywords:** agricultural non-point source pollution, field monitoring, nitrogen and phosphorus, paddy and maize, surface runoff

## Abstract

Surface runoff is one of the predominant routes for agricultural nitrogen (N) and phosphorus (P) losses, yet their characteristics and corresponding control measures are not fully understood. In 2019 and 2020, field-scale plot experiments were performed at Dongjiang Basin in South China to investigate the characteristics of N and P runoff losses from paddy and maize cropping systems. The results showed that N and P losses from maize fields *via* surface runoff (27.85 and 1.24 kg ha^−1^ year^−1^) were significantly higher than those from paddy fields (15.37 and 0.8 kg ha^−1^ year^−1^). The main forms of N losses were nitrate (${\text{NO}}_{3}^{-}$-N) and ammonium (${\text{NH}}_{4}^{+}$-N) in paddy and maize fields, respectively, whereas particulate P form predominated in surface runoff losses from both the paddy and maize fields. Considerable proportions of agricultural N and P (71–83% of the total runoff loss) were lost during basal fertilization and first topdressing application. Moreover, frequent rainfall events following fertilizer application triggered N and P losses from the monitored fields. About 26.22 and 37.48% of N fertilizer was recovered from grains and straw of paddy and maize, respectively, whereas only 12.35 and 19.51% of P fertilizer were recovered during the crop harvesting stage. Surface runoff was one of the dominant liquid pathways in N loss, whereas most of P loss (introduced from fertilizers without crops utilization) was fixed in the soil. Principal component analysis (PCA) proved that the primary sources of N and P losses were fertilizers rather than N and P in the soil. The current results suggest controlled management relating to fertilization, irrigation, and tillage strategies are effective measures for reducing N and P losses, thereby controlling agricultural non-point source pollution. It is hoped that this study will provide comprehensive field-based inputs on characteristics of N and P runoff losses and formulate appropriate control strategies to protect aquatic environments from eutrophication.

## Graphical Abstract

The experimental location and setup details. 
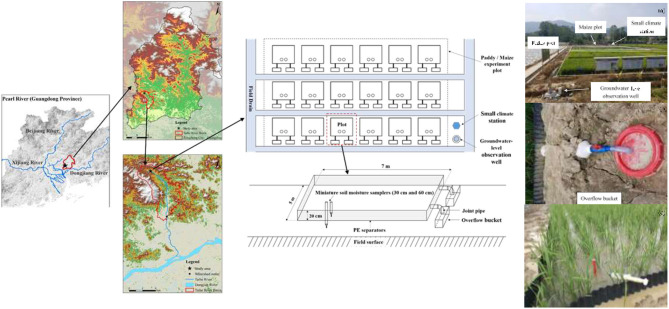

## Highlights

- The characteristics of N/P loss from crops were comprehensively studied in field.
- Rainfall after basal and first topdressing fertilization drove N/P runoff losses.
- Main N loss forms were ${\text{NO}}_{3}^{-}$-N (paddy) and ${\text{NH}}_{4}^{+}$-N (maize), while PP was main P form.
- Controlled fertilization, irrigation, and tillage can effectively reduce N/P loss.
- Surface runoff dominated in liquid N loss, and P tended to be fixed in soil.

## Introduction

In recent decades, chemical fertilizers containing nitrogen (N) and phosphorus (P) have been used extensively to achieve high crop yields. It was estimated that in 2017, the annual consumption of chemical N and P fertilizers in China increased to 29.6 and 12.3 million tons (Mt) according to the National Bureau of Statistic (2019), accounting for ~27.13 and 27.09% of global consumption of chemical N and P fertilizers (109.1 and 45.5 Mt), respectively (NBS National Statistics Bureau, 2019). However, the crop use efficiencies of N and P in China are reportedly below 30 and 20%, respectively (Xu et al., 2016). As a result, a considerable proportion of N and P is continuously released as agricultural non-point source (ANPS) pollution into the environment, leading to potential groundwater degradation (Ma et al., 2016) and contributing to surface water eutrophication (Le et al., 2010).

Quantifying agricultural N and P losses and elucidating their major pathways are essential for accurately estimating the environmental risks posed by ANPS pollution and formulating appropriate control strategies. Numerous recent studies have investigated N and P losses derived from fertilizer applications (Wang et al., 2014b; Ma et al., 2016; Hua et al., 2017). However, the characteristics and mechanisms of N and P losses are not fully understood. Agricultural losses in N and P are involved in complicated hydrological and biogeochemical processes, which can be influenced by many factors, such as climate, soil properties, and crop types (Zhan et al., 2020). Zhou et al. (2012) reported that N is mainly lost through ammonia volatilization, denitrification, surface runoff, and leaching processes. Of these processes, surface runoff is the predominant one contributing to N loss from both upland (Ma et al., 2016; Wang et al., 2019) and flooded soils (Liu et al., 2016). Some studies conducted on agricultural P losses have pointed to different pathways of P loss. For instance, Liu et al. (2016) found that runoff and leaching processes were two major routes for agricultural P losses, whereas Hua et al. (2017) found that P losses from fields predominantly occurred *via* runoff. Furthermore, the loss forms of N and P should be better understood, as they are crucial in selecting efficient approaches for controlling ANPS pollution (Gu et al., 2015).

The crop use efficiencies of agricultural N and P in China are relatively low from a global perspective. For instance, nitrogen use efficiency (NUE) values in China fall within the range of 20–40%, whereas the NUE values in the United States and Europe are as high as 65 and 61%, respectively (Liu et al., 2018, 2020). Thus, there is a considerable scope for improving crop use efficiencies of agricultural N and P in China, which, in turn, could reduce N and P losses that adversely affect the environment (Liu et al., 2018). Various controlled management strategies have been shown to be effective in improving the crop use efficiencies of N and P. These strategies can be broadly divided into three categories, namely, water-saving irrigation (Peng et al., 2015; Qi et al., 2020), fertilization especially for nitrogen management (Peng et al., 2017; Liu et al., 2020; Zhan et al., 2020), and sustainable conservation tillage modes (Issaka et al., 2019a). The application of water-saving irrigation has advantages in reducing water use and the likelihood of surface runoff discharge. It delays discharge time and decreases concentrated drainage pollutant loading during rainfall events by increasing water storage capacity in soil, thereby effectively controlling N and P losses *via* surface runoff (Peng et al., 2015; Zhuang et al., 2019). On the other hand, N and P losses are directly related to fertilizer inputs (Cui et al., 2018; Ding et al., 2020). An optimization of fertilization is beneficial on saving fertilizer usage, reducing N and P losses, and improving N and P use efficiencies (Cui et al., 2018; Qi et al., 2020). Moreover, conservation tillage possesses advantages over conventional tillage in controlling N and P losses through: (i) improving soil properties thereby decreasing nutrient mobility (Issaka et al., 2019a); (ii) reducing surface runoff velocity and increasing the retention time of the fertilizer in the top soil (Ali et al., 2007); and (iii) mitigating the extent of soil erosion by decreasing soil turbulence (Wang et al., 2019).

Dongjiang River is one of three major tributaries of the Pearl River in South China, and paddy and maize are two major crops cultivated in its basin area. As an important source of water supply of Guangdong province and Hong Kong, Dongjiang River is facing a potential deterioration in water quality because of intensive agricultural activities (Ding et al., 2016). Therefore, a series of field-scale plot experiments were established: (i) to monitor N and P losses through surface runoff from paddy and maize cropping systems at Dongjiang Basin, South China; (ii) to evaluate the main pathways and forms of N and P losses; and (iii) to investigate the effect of various fertilization, irrigation, and tillage-based management strategies on controlling N and P losses. This study entails a comprehensive evaluation of N and P losses and provides inputs for formulating an appropriate strategy for protecting the aquatic environment from eutrophication and deterioration.

## Materials and Methods

### Experimental Site and Soil Characteristics

**Figure 1:**
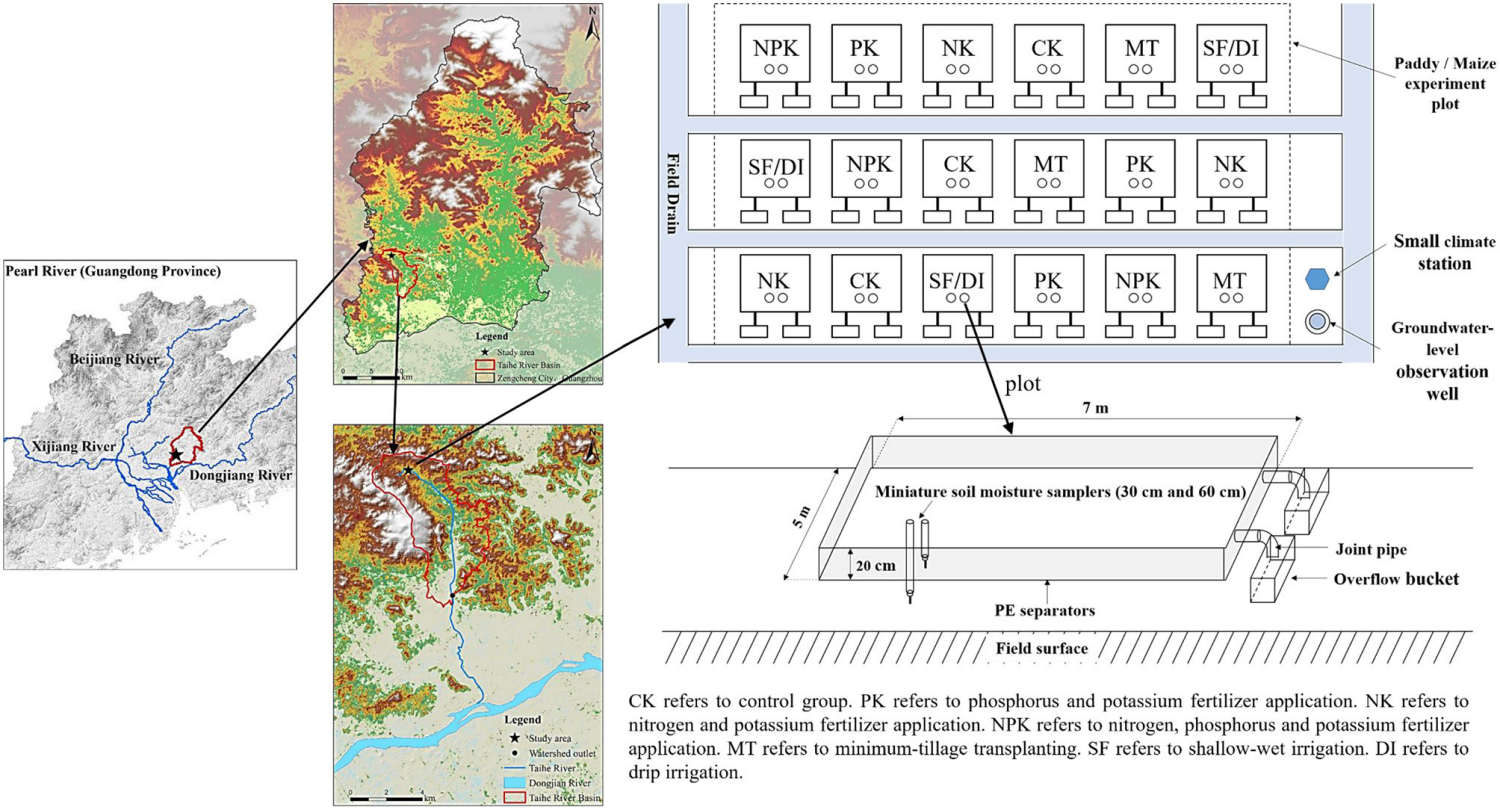
Location and layout of the study area.

Dongjiang Basin is a densely populated and intensively farm region with a high cropping index (i.e., the average number of annual crop seasons) in South China. For decades, chemical fertilizers were applied excessively in this region to achieve high crop yields and meet rapidly increasing demands for food. During the 2019–2020 period, we performed field-scale plot experiments in Ningxi town of Guangzhou, Guangdong province, China (23°14'N, 113°37' E). The study area is located in the lower reaches of Dongjiang River, along the western side (Figure 1). The study area has a precipitation ranging between 1,300 and 2,500 mm. Because of the typical subtropical monsoon climate, the temporal distribution of precipitation is uneven with around 70% of precipitation occurring from May to August (see Figure B1 in Appendix B).

The selected physicochemical properties of the topsoil (0–30 cm) in the study area are shown in Table A1 in Appendix A. The main soil type at Dongjiang Basin is acidic red soil, mainly derived from quaternary red parent material. The soil texture can be classified as sandy loam (14.6% clay, 15.9% silt, and 69.5% sand), which makes this soil type unfavorable for fertility retention, given the paucity of organic content (Chen et al., 2010). Other physicochemical characteristics of the topsoil are as follows: bulk density of 1.26 g cm^−3^, soil pH of 5.83, soil organic matter (SOM) of 15.49 g kg^−1^, total nitrogen (TN) of 1.05 g kg^−1^, and total phosphorus (TP) of 0.22 g kg^−1^, with available N and Olsen P of 60.97 g kg^−1^ and P 19.47 g kg^−1^, respectively.

### Field Experiment Design and Treatments

In this study, a total of 36 plots with an area of 35 m^2^ (5 × 7 m) for each plot were set up, which comprised six treatments for paddy fields and six treatments for maize fields in triplicates. As shown in Figure 1 and Figure B2 in Appendix B, each plot is separated using a PE separator that is inserted vertically 20 cm into the soil and 20 cm above the soil surface to avoid interference from other plots. A complete randomized block design was established for all the treatments and corresponding triplicates.

The species of paddy and maize that were selected in this study area were Huahang 51 and Yuetian 16, respectively. The detailed experimental setups are summarized in Table A2 in Appendix A. In brief, treatments for both paddy and maize included various methods of fertilization, irrigation, and tillage management. Three fertilizer practice groups were applied and included (i) conventional level of N (148 kg N ha^−1^), P (67 kg P ha^−1^), and potassium (K) (114 kg K ha^−1^) fertilizer applications as NPK group; (ii) conventional levels of P and K fertilizer applications but without N fertilizer as PK group; and (iii) conventional levels of N and K fertilizer applications but without P fertilizer as NK group. Urea [CO(NH_2_)_2_], mono calcium superphosphate [Ca(H_2_PO_4_)_2_], and potassium sulfate (KCl) were used as compound fertilizers (Table A2 in Appendix A). It should be noted that N fertilizer was applied with 30% of the total N as the basal application before seedlings were transplanted, and 40 and 30% of the total N were, respectively, applied in two topdressings 30 and 60 days after the seedlings had been transplanted. The above mentioned three fertilizer practice groups (i.e., NPK, PK, and NK) were performed with conventional tillage and conventional irrigation modes.

For the tillage practice groups, conventional tillage transplanting was applied by rotovating the soil at a depth of 20 cm followed by transplanting of 10-day-old paddy and maize seedlings. In comparison, conservation tillage of minimum tillage transplanting (MT) was applied by rotovating the soil at a depth of 10 cm followed by transplantation. It should be noted that either conventional tillage or conservation tillage practice groups were carried out with conventional fertilization and conventional irrigation modes (Table A2 in Appendix A).

For the irrigation practice groups, conventional irrigation management was applied, which refers to traditional flooding irrigation in accordance with the practices of local farmers. In the paddy fields, shallow-wet irrigation (SF) was applied as a water-saving irrigation mode. After establishing a shallow water layer, paddy was irrigated only when the water level fell to a certain depth. Drip irrigation was applied using surface drip lateral lines with self-compensating drippers (4 L h^−1^) as a mode of water-saving irrigation in the maize fields (DI). Conventional fertilization and tillage management modes were applied in all of the experimental irrigated groups (Table A2 in Appendix A). Lastly, for the control group (CK), neither fertilizer nor agronomic management practices (irrigation or tillage) were applied with conventional measures throughout the paddy and maize growing periods.

### Sample Collection and Analysis

As shown in Figure 1, two overflow buckets are constructed in parallel and connected to each plot with joint pipes to collect runoff and eroded soil particles after rainfall events. The total volume of the two overflow buckets was calculated based on the latest 2-year meteorological record for a single maximum precipitation value in the study area. Runoff water was thoroughly mixed in the overflow buckets and then collected from all the 36 plots immediately after each rainfall event. The runoff volume for each plot was recorded in field. The runoff water in the overflow buckets was pumped out with a vacuum pump after sample collection, and the volume of runoff water was recorded each time (Tables A3, A4 in Appendix A). In each plot, two miniature soil moisture samplers were inserted into the soil at depths of 30 and 60 cm, respectively. The leachate was sampled periodically at the two soil depths using a vacuum method (Issaka et al., 2019b). The groundwater level was also monitored throughout the study period by constructing a groundwater-level observation well near the experiment field. A small climate station was installed in order to automatically record precipitations during each rainfall event. The monitoring of N and P losses was carried out from August 2019 to July 2020, which encompassed two paddy growing seasons and three maize growing seasons. Detailed information of paddy and maize growing seasons in this study is summarized in Table A5 in Appendix A. Both runoff and leaching samples were collected after every rainfall event but only the volume of runoff water was quantified. All of the collected water samples were stored in 100-ml plastic bottles, transported to the laboratory, and stored at 4°C prior to being analyzed.

Soil samples were collected with a soil core sampler before planting commenced and right after crop harvest using an S-shaped, five-point collection method. The collection depths were 0–30 and 30–60 cm, which were consistent with the depths applied for the collection of leaching samples. At the harvesting stage of paddy and maize, crop samples were collected from a representative area of 1 m^2^ within each plot. Grain and straw were separated and weighed in field. The moisture content was also determined in field by weighing crop samples before and after they were air-dried. A total of five plant samples were randomly selected from each plot, and yield parameters were determined for paddy and maize, respectively. For paddy, these parameters were 1,000-grain weight and the seed-setting rate; and for maize, they were barren ear tips, ear rows, 100-grain weight, and the seed-producing rate of maize was determined accordingly. Analysis of other physicochemical properties and yield components were performed in the laboratory.

The TN concentration was determined for the collected samples using a Unico UV-2800 (Unicoi, USA) spectrophotometer following potassium peroxodisulfate digestion. Concentrations of ${\text{NH}}_{4}^{+}$-N and ${\text{NO}}_{3}^{-}$-N were analyzed with a continuous-flow analyzer (Skalar, Breda, the Netherlands), and the concentrations of TP on unfiltered samples and dissolved phosphorus (DP) on filtered samples (0.45 μm) were measured according to the ascorbic acid-molybdenum blue method (USEPA, 1979). The amount of particle-bound phosphorus (PP) was calculated as the difference between TP and DP.

The soil samples were air dried and sieved using mesh sizes of 2, 1, and 0.15 mm for laboratory analysis. Soil pH was measured using a glass electrode (pH meter) at a soil to solution ratio of 1:2.5. The SOM was measured using the dichromate oxidation method (Calvo-Fernandez et al., 2018). The soil TN content was analyzed using the Kjeldahl method (Jiao et al., 2018), while soil alkaline N (AN) content was analyzed using the alkaline hydrolysis method (Lu, 2000). Soil TP and Olsen P were determined using the ammonium molybdate method (Olsen and Sommers, 1982), after H_2_SO_4_-HClO_4_ digestion and 0.5 M NaHCO_3_ extraction.

All the plant samples were oven-dried until constant weight at 50°C, weighted, and then ground to pass through a 0.15-mm sieve. The subsamples were digested with H_2_SO_4_-H_2_O_2_ at 290°C. Thereafter, TN was determined using the Kjeldahl method, and TP was determined using the vanadium molybdate yellow colorimetric method (Lu, 2000).

### Calculation Methods

The depth of the ponding water collected in the overflow buckets was manually measured using a 1-m ruler immediately after every rainfall event. The runoff volume was calculated by multiplying the depth of the ponding water by the base area of the overflow buckets. The amount of TN or TP loss through surface runoff was calculated using Equation 1.

(1) $$\begin{array}{l} NRL=10^{4}\cdot \overset{n}{\underset{i=1}{\sum }}\left(Cn_{i}\cdot V_{i}\cdot 10^{-6}\right)/S\text{ or }\\ PRL=10^{4}\cdot \overset{n}{\underset{i=1}{\sum }}\left(Cp_{i}\cdot V_{i}\cdot 10^{-6}\right)/S \end{array}$$

where *NRL* and *PRL* denote losses in TN and TP, respectively, through surface runoff (kg ha^−1^year^−1^); *Cn*_*i*_ and *Cp*_*i*_ refer to TN and TP concentrations of runoff water in *I* sampling duration (mg L^−1^), respectively; *V*_*i*_ denotes the volume of surface runoff (L); *S* represents experimental plot area (35 m^2^); and *n* represents the number of samples collected in 1 year.

The crop use efficiencies of agricultural N and P are indicated by the ratio of N or P absorbed by grain and straw from fertilizer to the total amount of N or P applied during one growing season, and were calculated using Equations 2 and 3 below:

(2) $$\begin{array}{l} \text{N use efficiency}=\frac{NA_{NPK}-NA_{PK}}{A_{2}}\times 100\% \end{array}$$

(3) $$\begin{array}{l} \text{P use efficiency}=\frac{PA_{NPK}-PA_{NK}}{A_{2}}\times 100\% \end{array}$$

where *NA*_*NPK*_ and *PA*_*NPK*_ represent the total amount of N or P absorbed by the grain and straw of the NPK group, respectively; *NA*_*PK*_ denotes the amount of N absorbed by the grain and straw of the PK group; *PA*_*NPK*_ denotes the amount of P absorbed by the grain and straw of the NK group; and *A*_2_ is the total amount of N or P applied to the plot of the NPK group during one growing season (kg ha^−1^).

### Statistical Analysis

All measurements were performed in triplicates, and the data were presented as their arithmetic mean values. Data analysis was performed using SPSS statistics version 20.0. In each case, the data were statistically assessed by performing a one-way analysis of variance (ANOVA) with the minimum level of significance set at *p* < 0.05. PCA is a statistical technique that can be applied to transform a quantitative or qualitative dataset in an individual/variable table into a new set with a small number of variables or major components (Xu et al., 2019). PCA was performed on data compiled for runoff losses in TN, ${\text{NH}}_{4}^{+}$-N, ${\text{NO}}_{3}^{-}$-N, TP, PP, and DP for paddy and maize under six treatment conditions using the Origin 2017 program.

## Results

### Runoff Losses in Agricultural N From the Paddy Field

**Figure 2:**
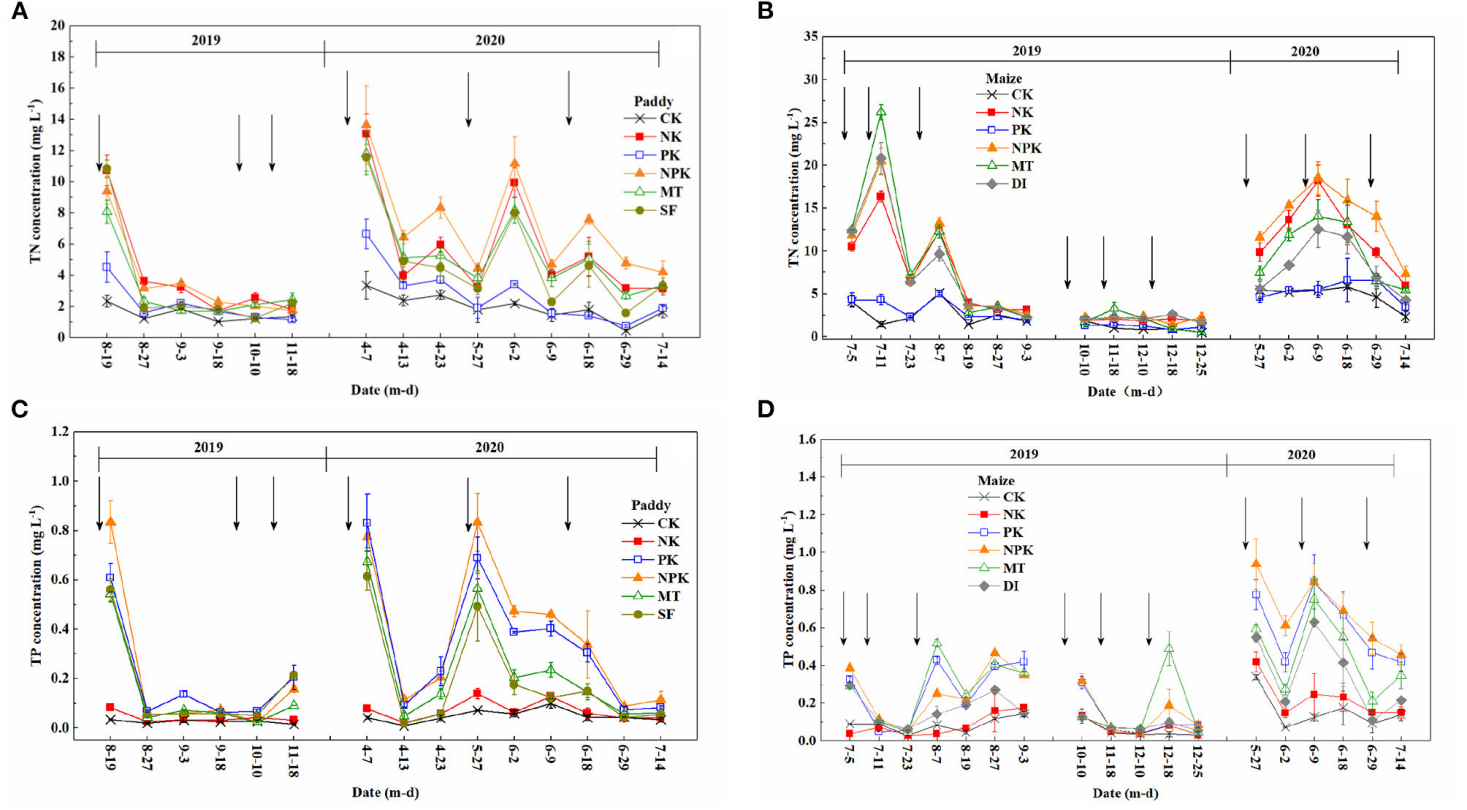
Total nitrogen concentration of runoff water in **(A)** paddy and **(B)** maize fields, total phosphorus concentration of runoff water in **(C)** paddy and **(D)** maize fields under different agricultural treatment conditions from July 2019 to July 2020. The symbol ↓ represents the time point of fertilizer application.

**Figure 3:**
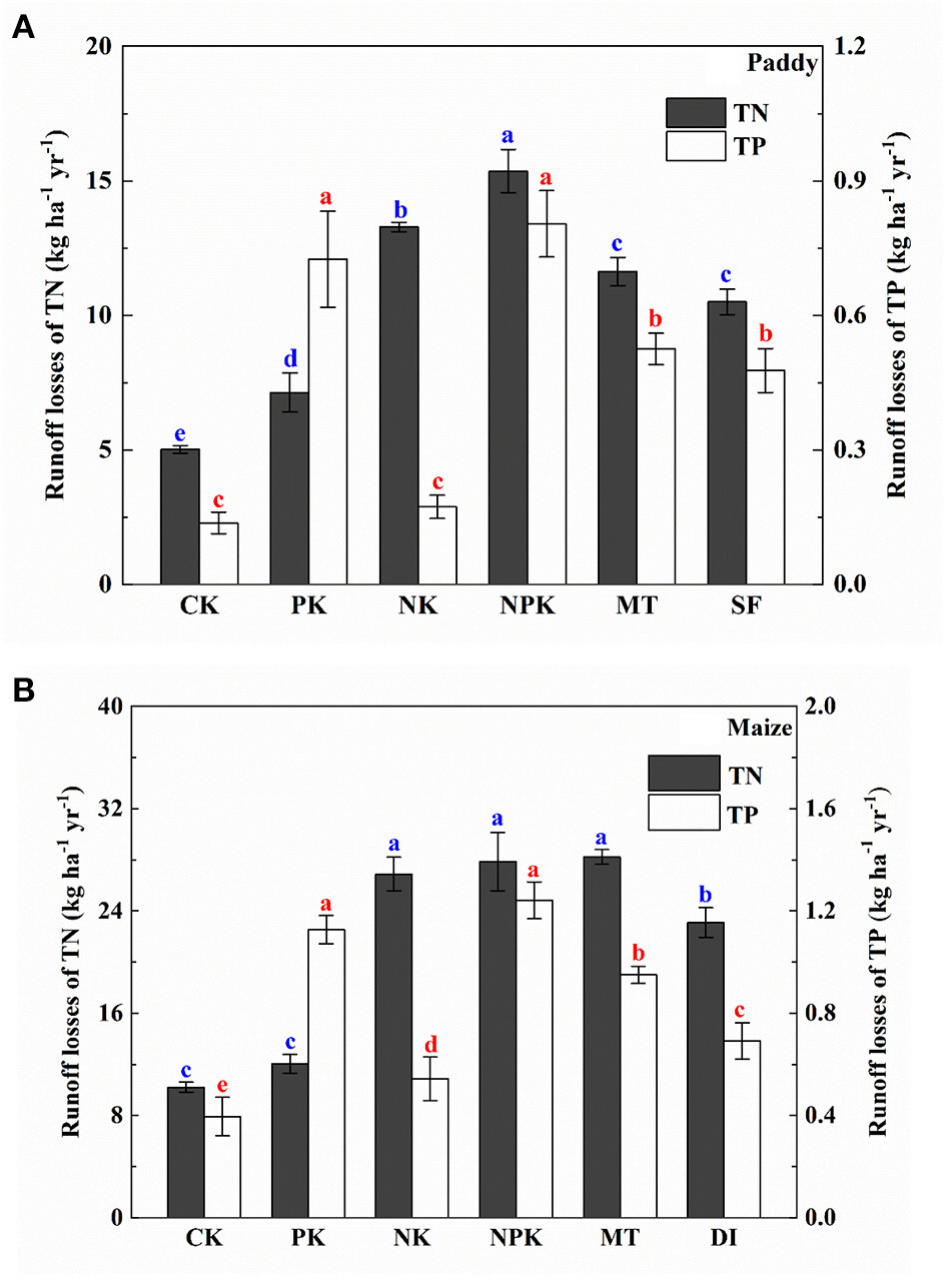
Runoff losses in total nitrogen (TN) and total phosphorus (TP) from **(A)** paddy and **(B)** maize fields under different agricultural treatment conditions in 1 year. Means within the same item followed by different letters are significantly different (LSD, *p* < 0.05).

**Figure 4:**
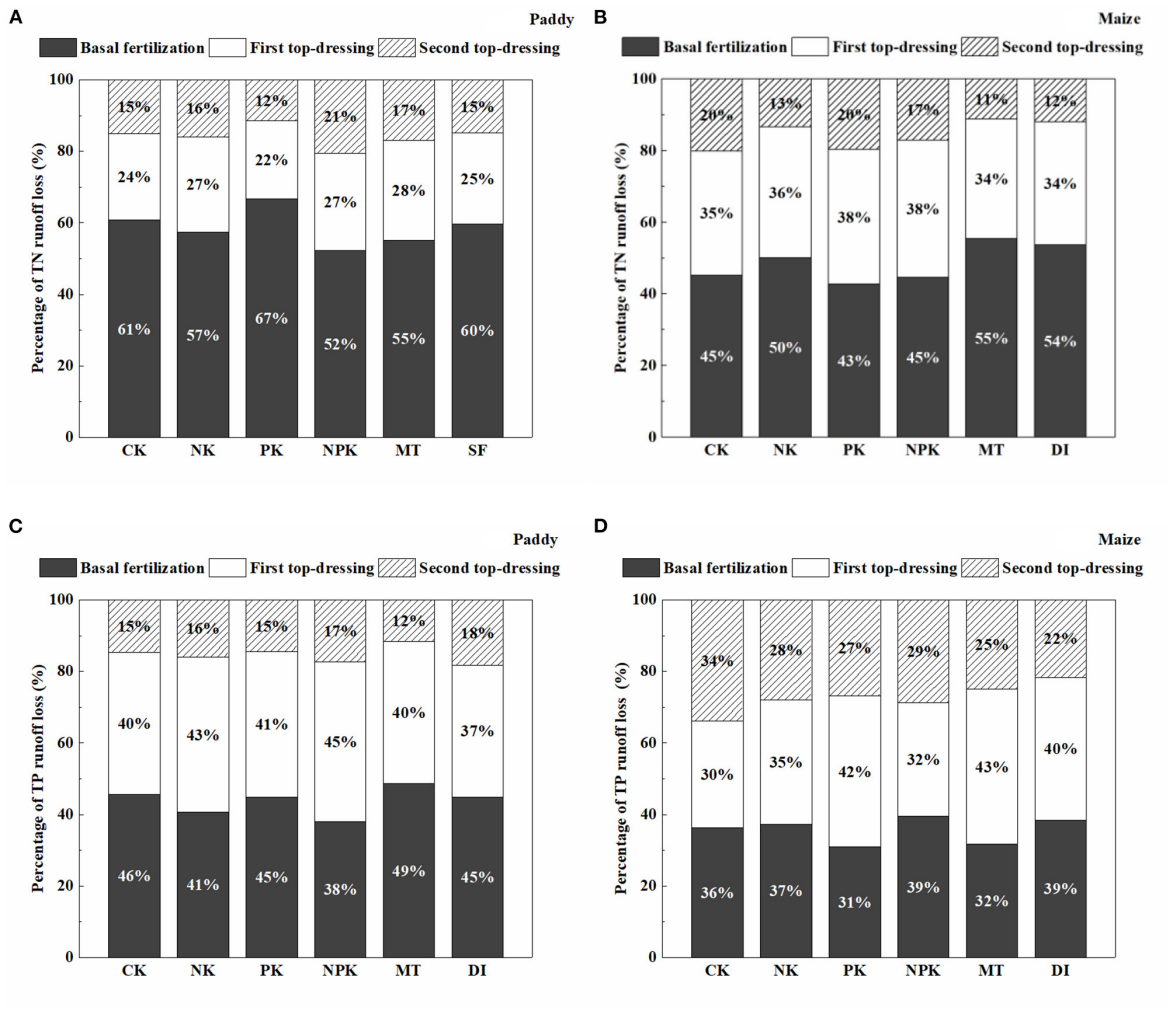
Percentage of total nitrogen (TN) runoff loss from **(A)** paddy and **(B)** maize fields, and percentage of total phosphorus (TP) runoff loss from **(C)** paddy and **(D)** maize fields during the basal fertilization stage, first top-dressing stage, and second top-dressing stage.

Figure 2A depicts the temporal dynamics of N surface runoff losses from the paddy field. The concentration of TN in the runoff water increased sharply right after fertilization and then gradually dropped until the next fertilization event in the paddy field. Peak TN concentrations were observed at the basal fertilization and first topdressing stages. The concentrations of TN ranged from 1.74 to 13.64 mg L^−1^ throughout the paddy-growing period for the NPK and NK groups, and were relatively higher than the concentrations for the non-N fertilized groups (i.e., PK and CK). Figure 3A shows the amount of runoff losses in TN from the paddy field with different agricultural treatments. Based on 1-year field monitoring, the highest agricultural N losses through surface runoff occurred in the NPK and NK groups (15.37 and 13.28 kg ha^−1^ year^−1^, respectively), whereas the amounts of TN losses *via* runoff in the PK and CK groups were 7.13 and 5.02 kg ha^−1^ year^−1^, respectively. As compared with the group with conventional tillage and irrigation mode (i.e., NPK), significantly lower TN concentrations were observed in the conservation tillage group (i.e., MT) and water-saving irrigation group (i.e., SF), in which TN concentrations in the SF group was even lower (assessed by one-way ANOVA test, *p* < 0.05). The amount of TN runoff losses in the MT and SF groups were 11.62 and 10.50 kg ha^−1^ year^−1^, respectively. The percentages of N runoff loss at different fertilization stages along paddy growing season were further calculated and shown in Figure 4a. It reveals that the percentages of N runoff loss during the basal fertilization stage were much higher than those during the two subsequent topdressing stages. Moreover, basal fertilization and the first topdressing contributed to 80% or more of the total runoff losses in all the treatment groups in the paddy field.

### Runoff Losses in Agricultural N From the Maize Field

Figure 2B shows the temporal dynamics of N surface runoff losses from the maize field. The TN concentrations from runoff water ranged between 1.45 and 20.45 mg L^−1^ in the NPK group. The peak TN concentration was determined during the first topdressing stage (Figure 2B). Throughout 1 year of monitoring, the higher N losses occurred in the NPK and NK groups, which is similar to that in the paddy field. As shown in Figure 3B, conventional agronomic fertilization and management led to a total loss of 27.85 kg ha^−1^ through surface runoff from the maize field over 1 year. Notably, the application of a mode of conservation tillage (i.e., MT) did not lower the amount of agricultural N runoff loss from the maize field, as compared with the conventional tillage method (i.e., NPK). By applying the DI mode, N loss *via* runoff was significantly lower than conventional irrigation mode with same level of N application (i.e., NPK, *p* < 0.05). Further calculation of percentages in N loss during different stages of fertilization revealed that N loss through surface runoff mainly occurred during the stages of basal fertilization and first topdressing, which accounted for more than 80% of the total runoff loss from the maize field.

### Agricultural P Losses Through Surface Runoff From the Paddy and Maize Fields

Figures 2C,D depict the temporal dynamics of P losses through surface runoff from the paddy and maize fields. In the paddy field, the TP concentrations in runoff water with a conventional fertilization mode (i.e., NPK) were in the range of 0.02–0.83 mg L^−1^ with peak TP values occurring during the basal fertilization and first topdressing stages. The TP concentrations in the PK group were comparable with those in the NPK group during the paddy-growing period. As compared with conventional management regimes, concentrations of TP loss though surface runoff were reduced under controlled management regimes of MT and SF. As shown in Figure 3A, annual P runoff losses in the MT and SF groups were 0.53 and 0.48 kg ha^−1^ year^−1^, respectively, which were significantly lower than those under conventional tillage and irrigation modes (0.8 kg ha^−1^ year^−1^ in the NPK group). The percentage of P loss was more than 80% of the total P runoff loss, calculated by summing P losses during the basal fertilization and first topdressing stages. The P runoff loss during these two stages were almost equal (Figure 4C).

Peak concentrations of P loss through surface runoff in the maize field were generally detected during the stages of basal fertilization and first topdressing, with the exception of the MT and DI groups in 2019. As shown in Figure 3B, the total losses of P through surface runoff were 1.24 and 1.13 kg ha^−1^ year^−1^ in the NPK and PK groups, respectively. The P losses were lower in the NK and CK groups, as expected, as no application of P fertilizer was applied throughout the maize-growing period. Similar with the paddy group, annual P runoff losses from the maize field in the MT and DI groups (0.95 and 0.69 kg ha^−1^ year^−1^, respectively), were significantly lower than those in conventional management (*p* < 0.05).

### Residual Soil Content of N and P After Crop Harvest

After crop harvest, soil samples collected from depths of 0–30 cm (the topsoil layer) and 30–60 cm (the subsoil layer) were further analyzed. Compared with the CK and PK groups, the soil content of TN at depths of 0–30 cm was 1.34–1.4 g kg^−1^ in the paddy field and 1.1–1.21 g kg^−1^ in the maize field, which was obviously higher in all the treatment groups with N fertilizer applied (Figure B4a in Appendix B). Meanwhile, the soil content of TN at the depth of 30–60 cm was also higher in the N fertilizer-applied groups regardless of irrigation and tillage measures. This result suggests that, apart from N losses through surface runoff, N also migrated downward though a leaching process, as confirmed by the TN concentration detected in leachates obtained at the above-described soil depths (Figures B6a,c in Appendix B). As for available N in soil, a relatively higher content was detected in the topsoil of the SF group (81.53 mg kg^−1^) in the paddy field and the MT group (94.59 mg kg^−1^) in the maize field among the treatment groups with application of N fertilizer (Figures B4b, B5b in Appendix B). Similar to the tendency of TN, the soil TP content increased in both the topsoil and subsoil layers after the application of P fertilizer, ranging from 0.76–1.44 and 0.93–1.27 g kg^−1^ in the paddy and maize fields, respectively (Figures B4c, B5c in Appendix B). Considering the dynamics of TP concentrations in leachates (Figures B6b, B6d in Appendix B), we posit that the leaching process is likely to be another route for losses of agricultural P. Controlled managements of conservation tillage (i.e., MT) and water-saving irrigation (i.e., SF and DI) did not significantly affect the Olsen P content of the topsoil and subsoil (Figure B5d in Appendix B). In both the paddy and maize fields, SOM values were slightly higher in all the fertilized groups than that in CK (Figures B4f, B5f in Appendix B). Furthermore, the soil pH of the topsoil and subsoil layers in the paddy field was 6.22–6.29 and 5.6–5.92, respectively; while the soil pH of the topsoil and subsoil layers in the maize field was 5.59–5.76 and 5.45–5.64, respectively (Figure B5e in Appendix B). The relatively lower pH values recorded for the soil in the maize field are likely to have been caused by the larger amount of N fertilizer applied to maize (849 kg ha^−1^ year^−1^) compared with applications in the paddy field (668 kg ha^−1^ year^−1^). Moreover, a higher N input could enhance soil acidification by accelerating base cation losses from the soil (Zhu et al., 2018).

### Crop Yields and N/P Use Efficiencies

The grain yields of paddy and maize under conventional fertilization and management (i.e., NPK) were 5023.79 and 8258.84 kg ha^−1^, respectively (Tables 1, 2). We found that the grain yield was as low as 2,021.44 kg ha^−1^ in the unfertilized paddy field (i.e., CK) and no crop yields were recorded in this group, suggesting the crucial role of fertilizer in achieving high crop yields. Furthermore, the grain yields of both paddy and maize in the NPK groups were comparable with yields in the NK group lacking fertilizer application, but they were significantly higher than those in the PK group without N fertilizer application (*p* < 0.05). These results are quite consistent with the growing status of paddy and maize observed during their harvesting stages (Figure B3 in Appendix B). Their growing status in the NPK and NK groups appeared similar, but clearly differed from the status of paddy and maize in the PK group. Furthermore, as shown in Tables 1, 2, the N and P content was relatively higher in maize grain (21.82 and 5.25 g kg^−1^, respectively), than in paddy grain (12.6 and 1.48 g kg^−1^, respectively). It is expected that the content of N and P in grain and straw would correspondingly decrease without applications of N and P fertilizers. The analysis of agronomic traits showed that 1,000-grain weight and seed-setting rate of paddy were 21.31 g and 91.33%, respectively. Measures for maize, notably barren ear tips, the kernel row number, the 100-grain weight, and the seed-setting rate were 2.40 cm, 34, 35.29 g, and 75.02%, respectively. The effects of fertilization application, especially N fertilizer application, on agronomic traits were found to be significant (Tables 1, 2).

Table 1Agronomic traits and fertilizer utilization rate of paddy under different agriculture treatment conditions.

**Table:** 

**Crop type**	**Treatment**	**Grain yield**	**Straw yield**	**1,000-grain weight**	**Seed-setting rate**
		**(kg ha^**−1**^)**	**(kg ha^**−1**^)**	**(g)**	**(%)**
Paddy	CK^a^	2021.44 ± 235.68^c^	5278.06 ± 294.35^c^	20.77 ± 0.12^b^	87.00 ± 1.08^b^
	PK^b^	4176.16 ± 81.93^b^	8850.00 ± 764.24^b^	21.82 ± 0.51^a^	86.83 ± 3.27^b^
	NK^c^	5176.21 ± 326.18^a^	10350.00 ± 421.36^a^	21.11 ± 0.38^a^^b^	91.17 ± 2.46^a^
	NPK^d^	5023.79 ± 152.24^a^	10250.00 ± 535.76^a^^b^	21.31 ± 0.38^a^^b^	91.33 ± 0.71^a^
	MT^e^	5385.71 ± 414.73^a^	10708.00 ± 486.00^a^	21.11 ± 0.31^a^^b^	93.00 ± 1.47^a^
	SF^f^	5380.96 ± 291.74^a^	11409.50 ± 1433.19^a^	21.41 ± 0.22^a^^b^	93.00 ± 1.08^a^

**Table:** 

**Crop type**	**Treatment**	**Grain TN**	**Grain TP**	**Straw TN**	**Straw TP**	**N use efficiency**	**P use efficiency**
		**(g kg** ^**−1**^ **)**	**(g kg** ^**−1**^ **)**	**(g kg** ^**−1**^ **)**	**(g kg** ^**−1**^ **)**	**(%)** ^**h**^	**(%)** ^**i**^
Paddy	CK^a^	11.22 ± 0.28^b^	1.22 ± 0.10^b^	8.13 ± 0.34^c^	0.90 ± 0.14^b^	/^j^	/
	PK^b^	11.63 ± 0.30^b^	1.47 ± 0.03^a^	9.30 ± 0.66b^c^	1.24 ± 0.08^a^	/	/
	NK^c^	13.43 ± 0.51^a^	1.31 ± 0.07^b^	10.42 ± 0.04^b^	0.89 ± 0.25^b^	/	/
	NPK^d^	12.60 ± 0.69^a^^b^	1.48 ± 0.18^a^	10.38 ± 0.51^b^	1.10 ± 0.18^a^^b^	26.22 ± 0.88^b^	12.35 ± 0.97^a^
	MT^e^	13.56 ± 0.40^a^	1.25 ± 0.13^b^	12.43 ± 1.18^a^	1.15 ± 0.09^a^^b^	29.81 ± 1.08^a^	14.08 ± 0.14^a^
	DI^g^	12.58 ± 0.08^a^^b^	1.33 ± 0.06^b^	11.30 ± 1.55^a^^b^	1.16 ± 0.15^a^^b^	30.43 ± 2.18^a^	13.78 ± 0.43^a^

a *CK refers to control group*.

b *PK refers to phosphorus and potassium fertilizer application*.

c *NK refers to nitrogen and potassium fertilizer application*.

d *NPK refers to nitrogen, phosphorus, and potassium fertilizer application*.

e *MT refers to minimum tillage transplanting*.

f *SF refers to shallow-wet irrigation*.

g *DI refers to drip irrigation*.

h *The paddy N use efficiency was calculated from the difference in the amount of N absorbed by grain and straw between NPK group and PK group in relation to the total amount of N applied, based on the stoichiometry of Eq. 2. The amount of N absorbed by grain and straw consists of grain yield multiplied by grain TN concentration and straw yield multiplied by straw TN concentration*.

i *The paddy P use efficiency was calculated from the difference in the amount of P absorbed by grain and straw between NPK group and NK group in relation to the total amount of P applied, based on the stoichiometry of Eq. 3. The amount of P absorbed by grain and straw consists of grain yield multiplied by grain TP concentration and straw yield multiplied by straw TP concentration*.

j */refers to index that cannot be calculated*.

*Means (n = 3) within a column followed by different letters are significantly different (LSD, p < 0.05)*.

Table 2Agronomic traits and fertilizer utilization rate of maize under different agriculture treatment conditions.

**Table:** 

**Crop type**	**Treatment**	**Grain yield**	**Straw yield**	**Barren ear tip**	**Kernel row**	**100-grain weight**	**Seed-producing rate**
		**(kg ha^**−1**^)**	**(kg ha^**−1**^)**	**(cm)**	**number**	**(g)**	**(%)**
Maize	CK^a^	0^c^	2800.22 ± 126.54^d^	/	0^c^	0^c^	0^c^
	PK^b^	5133.58 ± 564.81^b^	6939.32 ± 477.61^c^	3.0 ± 0.85^a^^b^	26 ± 1^b^	25.45 ± 4.50^b^	66.29 ± 6.93^b^
	NK^c^	7870.72 ± 13.84^a^	7539.70 ± 100.50^b^^c^	3.35 ± 0.42^a^	32 ± 1^a^	30.27 ± 6.55^a^^b^	74.57 ± 1.86^a^
	NPK^d^	8158.84 ± 500.56^a^	8104.20 ± 300.46^a^^b^	2.40 ± 0.12^b^	34 ± 2^a^	35.29 ± 2.73^a^	75.02 ± 0.53^a^
	MT^e^	8059.10 ± 956.92^a^	8764.79 ± 196.55^a^	2.65 ± 0.32^a^^b^	32 ± 2^a^	33.24 ± 1.13^a^	72.82 ± 2.22^a^^b^
	SF^f^	7773.17 ± 126.36^a^	8014.08 ± 688.55^a^^b^	2.37 ± 0.40^a^^b^	33 ± 1^a^	28.99 ± 1.14^a^^b^	71.17 ± 1.14^a^^b^

**Table:** 

**Crop type**	**Treatment**	**Grain TN**	**Grain TP**	**Straw TN**	**Straw TP**	**N use efficiency**	**P use efficiency**
		**(g kg** ^**−1**^ **)**	**(g kg** ^**−1**^ **)**	**(g kg** ^**−1**^ **)**	**(g kg** ^**−1**^ **)**	**(%)** ^**h**^	**(%)** ^**i**^
Maize	CK^a^	18.73 ± 1.02^c^	2.06 ± 0.10^b^	15.11 ± 0.17^b^	4.96 ± 0.07^a^^b^	/^g^	/
	PK^b^	20.96 ± 0.40^b^	2.40 ± 0.03^a^	16.26 ± 1.00^b^	5.41 ± 0.13^a^	/	/
	NK^c^	23.98 ± 0.35^a^	2.10 ± 0.04^b^	23.14 ± 2.04^a^	4.56 ± 0.20^b^	/	/
	NPK^d^	24.77 ± 1.24^a^	2.48 ± 0.12^a^	21.82 ± 2.36^a^	5.25 ± 0.21^a^	37.48 ± 2.55^a^	19.51 ± 1.06^a^
	MT^e^	25.59 ± 1.10^a^	2.56 ± 0.11^a^	24.00 ± 0.48^a^	5.30 ± 0.36^a^	40.33 ± 0.85^a^	20.24 ± 0.47^a^
	DI^g^	24.04 ± 1.46^a^	2.40 ± 0.15^a^	21.64 ± 3.93^a^	5.19 ± 0.18^a^	41.86 ± 1.60^a^	20.56 ± 0.47^a^

a *CK refers to control group*.

b *PK refers to phosphorus and potassium fertilizer application*.

c *NK refers to nitrogen and potassium fertilizer application*.

d *NPK refers to nitrogen, phosphorus, and potassium fertilizer application*.

e *MT refers to minimum tillage transplanting*.

f *SF refers to shallow-wet irrigation*.

g *DI refers to drip irrigation*.

h *The maize N use efficiency was calculated the same as paddy*.

i *The maize P use efficiency was calculated the same as paddy*.

j */refers to index cannot be calculated*.

*Means (n = 3) within a column followed by different letters are significantly different (LSD, p < 0.05)*.

Under controlled managements of conservation tillage and water-saving irrigation modes, the grain yields and other agronomic traits were found yet insignificantly affected in both the paddy and maize fields. However, the use of either conservation tillage or water-saving irrigation could result in higher crop use efficiencies of N and P compared with those obtained using conventional management regimes. Furthermore, it is noteworthy that the N and P content in both the paddy and maize grains exceeded that of the grains obtained in the groups applying conservation tillage and water-saving irrigation. These results are in line with higher crop use efficiencies of N and P resulting from controlled management.

### PCA Analysis

PCA of agricultural N and P runoff losses from paddy and maize fields was performed with different agricultural treatment modes (i.e., fertilization, irrigation, and tillage). As shown in **Figure 6**, the two principal components explain 91.7 and 91.2% of variabilities based on the monitored data from the paddy and maize fields, respectively. The first principal component (PC1 axis), which accounted for 59.6 and 55.9% in the paddy and maize groups, respectively, represents the total amount of N and P losses through surface runoff associated with different agricultural treatments (Table A6 in Appendix A). The NPK groups were scattered and oriented in the positive direction of the PC1 axis, with the same directions evident for total N and P losses as well as for their different forms. However, the distribution of the CK group was aligned with the negative direction of the PC1 axis (**Figure 6**), indicating the lowest amount of N and P losses in that group (with no fertilizer applied). The second principal component (PC2 axis) explained 32.1 and 35.3% of the total variance in N and P losses from the paddy and maize fields, respectively. The PK group without N fertilizer and the NK group without P fertilizer were distributed distantly from each other along the PC2 axis. The distribution of the PK group was scattered in alignment to the positive direction of the PC2 axis with the same directions of P loss evident for differing forms. The distribution of the NK group was scattered in alignment with the negative direction of the PC2 axis in the same direction of N loss.

## Discussion

### Characteristics of Agricultural N and P Losses *via* Surface Runoff From Paddy and Maize Fields

Surface runoff process is one of the main routes contributing to agricultural N and P losses and, consequently, severe ANPS pollution (Ma et al., 2016; Hua et al., 2017; Wang et al., 2019). In this study, 1-year monitoring was carried out to investigate the characteristics of N and P surface runoff losses from a flooded field (i.e., paddy) and an upland field (i.e., maize). The results demonstrated that agricultural N and P losses *via* surface runoff from the paddy field were 15.37 and 0.8 kg ha^−1^ year^−1^, respectively, which encompassed two paddy growing seasons, whereas those from the maize field were 27.85 and 1.24 kg ha^−1^ year^−1^, respectively, which encompassed three maize growing seasons. A previous study reported that maize was the most N-polluting of the five crops investigated (Malik and Dechmi, 2020). Wang et al. (2019) summarized 56 published studies that performed in 13 Chinese provinces. Ranges of 0.43–88.2 kg ha^−1^ of seasonal N runoff losses and 0.01–12.6 kg ha^−1^ of seasonal P runoff losses from the paddy fields as well as ranges of 0.18–49.5 kg ha^−1^ and 0.01–16.4 kg ha^−1^ of N and P runoff losses, respectively, were reported (Wang et al., 2019). Peak concentrations of TN and TP runoff losses were 13.64 mg L^−1^ and 0.83 mg L^−1^, respectively, in the paddy field, but can go as high as 26.16 mg L^−1^ and 0.94 mg L^−1^, respectively, in the maize field. This result is consistent with that of a previous study in which the TP concentration in the agricultural runoff can reach as high as 22.4 and 1.2 mg L^−1^ during storm events (Hua et al., 2017; Issaka et al., 2019b). The detected concentrations of TN and TP were above 0.66 and 0.05 mg L^−1^, respectively, thus exceeding reference values for both TP and TN provided by the USEPA (2000), and could lead to eutrophication. The results suggest that runoff water from Dongjiang Basin could contribute to ANPS pollution if it is directly discharged into the water body. Furthermore, the stages of basal fertilization and first topdressing were found to contribute 71–83% of the TN and TP losses in surface runoff from both the paddy and maize fields in the NPK groups. To the knowledge of the authors, this is one of the few studies that quantitatively identify “potential risk period” of N/P losses (basal fertilization and the first topdressing stages) during paddy and maize growing seasons at Dongjiang Basin. Moreover, peak concentrations of N and P losses were observed immediately after these two fertilization events. One possible reason could be the relatively low crop use efficiencies of N and P during the early growing stages of the crops. On the other hand, it is noteworthy that higher concentrations of N and P losses were generally detected from May to August, when heavy rainfall events occurred frequently and precipitation accounted for ~70% of the total annual precipitation. The relationship between precipitation and runoff loss in TN and TP was tentatively established and shown in Figure B7 in Appendix B. Correlation analysis indicates that there is a significant and positive relationship between precipitation and either N or P loss in both the paddy and maize fields (*p* < 0.01). The obtained result is in line with previous studies carried out in other regions such as Chaohu region and Wuchuan catchment in China (Cao et al., 2014; Wang et al., 2014a). N and P originated from fertilization flushed out by runoff during rainfall events likely because N and P had not yet accumulated in the soil. According to the theory of “infiltration-excess,” surface runoff becomes significant when rainfall intensity exceeds the infiltration capacity of soil (Horon, 1933). Fu et al. (2019) found that the highest proportion of N runoff losses occurred when seasonal precipitation exceeded 500 mm in South China. Based on a more recent study that monitored for 30 years N loss in the rice-wheat cropping rotation system, N losses in the period with both frequent rainfall events and intensive fertilization (i.e., June to August) accounted for 46.4–54.5% of total annual N losses (Diao et al., 2020). Interestingly, it is also reported in this study that N losses in this period was found to mainly occur *via* the runoff process, whereas both runoff and leaching were significant during other periods (Diao et al., 2020). As a result, frequent rainfall event following fertilizer application is the key factor driving N and P losses from cropping systems *via* the surface runoff process.

The forms of N and P loss *via* surface runoff from the paddy and maize fields were further investigated and are shown in Table 3. The analysis revealed that ${\text{NO}}_{3}^{-}$-N was the main form of N loss from the paddy field, accounting for 44.4–55.83% of TN losses. On the contrary, ${\text{NH}}_{4}^{+}$-N was the predominant form of N loss from the maize field, accounting for 50.27–62.32% of TN losses. This is likely caused by lack of nitrate reductase in the roots of paddy rice, leading to inefficient utilization of ${\text{NO}}_{3}^{--}$N (Fan et al., 2007). Conversely, upland crops, such as maize, tend to absorb ${\text{NO}}_{3}^{--}$N rather than ${\text{NH}}_{4}^{+}$-N as the main source of N. The runoff losses of P included particulate and dissolved forms. Based on the monitoring result, PP was the dominant form of P runoff loss from both the paddy and maize fields. This finding is in line with that of a previous study that revealed PP accounted for 60–75% of TP in the Chaohu Lake region (Wang et al., 2014a). Moreover, frequent precipitation may accelerate the loss of particulate nutrients by facilitating the erosion of topsoil in upland fields (Ma et al., 2016). This finding may explain why the environmental risk of P loss *via* surface runoff was greater for the maize cropping system than for the paddy cropping system.

**Table 3:** Surface runoff losses of ammonium (${\text{NH}}_{4}^{+}$-N), nitrate (${\text{NO}}_{3}^{-}$-N), particle-bound phosphorus (PP), and dissolved phosphorus (DP) from paddy and maize fields.

**Crop type**	**Treatment**	${\text{NH}}_{4}^{+}$ **-N**		${\text{NO}}_{3}^{-}$ **-N**		**PP**		**DP**	
		**Loss**	**Percentage of**	**Loss**	**Percentage of**	**Loss**	**Percentage of**	**Loss**	**Percentage of**
		**(kg ha^**−1**^)**	**TN loss (%)**	**(kg ha^**−1**^)**	**TN loss (%)**	**(kg ha^**−1**^)**	**TP loss (%)**	**(kg ha^**−1**^)**	**TP loss (%)**
Paddy	CK^a^	1.21 ± 0.25^c^	23.93 ± 3.17^a^	2.43 ± 0.40^e^	48.15 ± 4.72^a^	0.10 ± 0.03^c^	70.04 ± 7.29^a^	0.05 ± 0.01^a^	33.78 ± 2.63^a^
	PK^b^	1.37 ± 0.06^c^	19.33 ± 2.53^ab^	3.65 ± 0.53^de^	55.83 ± 0.78^a^	0.57 ± 0.11^c^	77.81 ± 3.09^a^	0.16 ± 0.00^a^	22.46 ± 2.91^b^
	NK^c^	2.75 ± 0.26^a^	20.75 ± 1.54^ab^	7.19 ± 0.35^b^	50.88 ± 3.84^a^	0.15 ± 0.04^a^	84.99 ± 0.17^a^	0.05 ± 0.01^b^	28.99 ± 2.98^ab^
	NPK^d^	3.12 ± 0.16^a^	20.45 ± 2.45^ab^	8.57 ± 0.55^a^	54.44 ± 5.50^a^	0.63 ± 0.08^a^	78.45 ± 3.62^a^	0.17 ± 0.03^b^	21.55 ± 3.62^b^
	MT^e^	2.03 ± 0.08^b^	17.52 ± 0.89^b^	5.12 ± 0.29^c^	44.40 ± 5.03^a^	0.3/ ± 0.03b^c^	71.86 ± 0.42^a^	0.12 ± 0.01^a^	23.73 ± 2.62^b^
	SF^f^	2.00 ± 0.27^b^	19.04 ± 2.78^ab^	4.68 ± 0.49^cd^	44.55 ± 4.39^a^	0.30 ± 0.04^c^	63.20 ± 8.15^a^	0.17 ± 0.05^a^	30.08 ± 0.46^a^
Maize	CK^a^	5.90 ± 0.31^d^	54.90 ± 2.95^ab^	2.59 ± 0.32^a^	25.42 ± 3.53^a^	0.28 ± 0.06^d^	69.79 ± 3.14^ab^	0.12 ± 0.02^b^	30.21 ± 3.14^ab^
	PK^b^	6.03 ± 0.12^d^	50.27 ± 3.39^b^	2.90 ± 0.39^b^	23.99 ± 2.43^ab^	0.87 ± 0.05^ab^	77.52 ± 2.15^ab^	0.25 ± 0.03^ab^	22.48 ± 2.15^ab^
	NK^c^	16.07 ± 1.25^ab^	59.92 ± 5.76^a^	5.45 ± 0.25^a^	20.34 ± 1.61^ab^	0.35 ± 0.02^d^	66.98 ± 5.92^b^	0.19 ± 0.01^ab^	33.02 ± 3.52^a^
	NPK^d^	16.99 ± 0.57^a^	61.23 ± 2.89^a^	5.59 ± 0.43^b^	20.13 ± 1.44^ab^	0.98 ± 0.08^a^	78.72 ± 3.84^ab^	0.26 ± 0.05^a^	21.28 ± 3.40^ab^
	MT^e^	17.17 ± 1.67^a^	60.80 ± 5.66^a^	5.08 ± 0.26^a^	18.01 ± 1.28^b^	0.78 ± 0.04^b^	82.52 ± 7.08^a^	0.17 ± 0.07^ab^	17.48 ± 4.32^b^
	DI^g^	14.38 ± 1.31^b^	62.32 ± 5.20^a^	5.28 ± 0.19^a^	22.95 ± 1.90^ab^	0.53 ± 0.07^c^	75.68 ± 2.56^ab^	0.17 ± 0.01^ab^	24.32 ± 2.56^ab^

a *CK refers to control group*.

b *PK refers to phosphorus and potassium fertilizer application*.

c *NK refers to nitrogen and potassium fertilizer application*.

d *NPK refers to nitrogen, phosphorus, and potassium fertilizer application*.

e *MT refers to minimum tillage transplanting*.

f *SF refers to shallow-wet irrigation*.

g *DI refers to drip irrigation*.

*Means (n = 3) within a column followed by different letters are significantly different (LSD, p < 0.05)*.

### Exploration of Main Pathways of Agricultural N and P Imported From Fertilizer Within Different Cropping Systems

**Figure 5:**
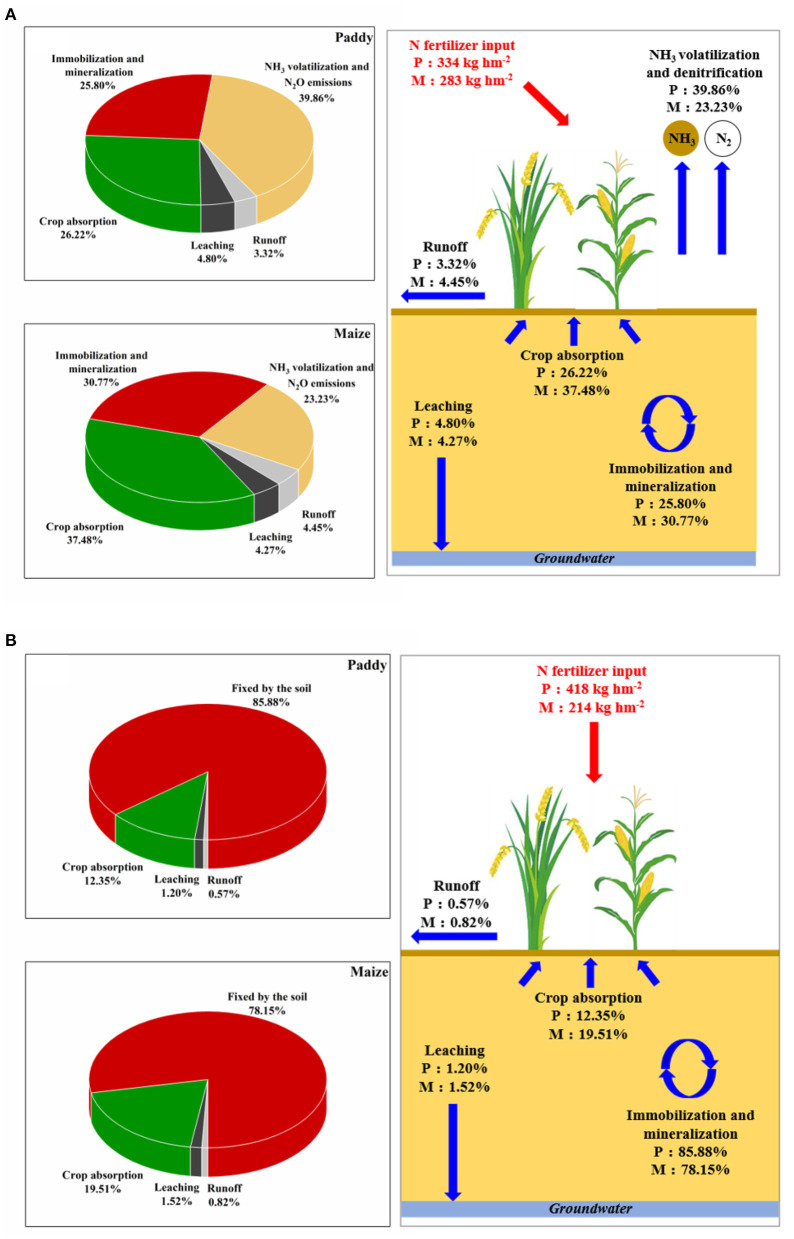
Possible cycling pathways of **(A)** nitrogen (N) and **(B)** phosphorus (P) in paddy fields and maize fields.

The main pathways of agricultural N and P imported from fertilizer were tentatively explored and shown in Figure 5. The NUE calculations indicated that 26.22 and 37.48% of N fertilizer was recovered from the grains and straw of paddy and maize after crop harvests (Tables 1, 2). Zhang et al. (2008) reported a China-wide range of NUE values (26.1–28.3%) for three major crops (i.e., paddy, wheat, and maize). By measuring soil TN content at depths of 30 and 60 cm, it is roughly estimated that 26.36–30.77% of N was immobilized or mineralized by soil particulates and microbes. Approximately 47.98% of the remaining N fertilizer was lost from the paddy field. A lower proportion of agricultural N loss (31.75%) was observed in the maize field. It was generally considered that N was lost mainly in gaseous and liquid forms. Here, we referred to estimation values derived from a previous study conducted in China, which reported that 23.23–42.5% of agricultural N was lost through gaseous forms (i.e., NH_3_, N_2_, NO, and N_2_O emissions) *via* ammonia volatilization and denitrification processes (Ding et al., 2020). Thus, the total proportion of N loss in liquid forms was estimated to be around 8%. According to the field monitoring, the proportions of N fertilizer losses *via* surface runoff were 3.32 and 4.45% in the paddy and maize fields, respectively. The remaining 4.8 and 4.27% of N fertilizer was likely lost *via* leaching and subsurface runoff processes.

As for the pathway of the agricultural P derived from fertilizer, it can be seen from Figure 5B that 12.35 and 19.51% of P fertilizer was, respectively, recovered from paddy and maize cropping systems during the harvesting stage. The crop use efficiency of P was lower than that of N in corresponding cropping system. Zhang et al. (2008) reported respective ranges of 11.6–13.7 and 9.7–12.6% of P use efficiency in paddy and maize cropping systems across China. The results notably indicate that large proportions of P were fixed in soil particulate in the paddy field (85.88%) and the maize field (78.15%). This is likely due to the high content of Fe and Al in the red soil in South China, and Fe and Al have strong capacities for binding P in red soil; thus, PP was the predominant P form in the soil (Hua et al., 2017). The proportions of P fertilizer loss *via* surface runoff were 0.57 and 0.82% in the paddy and maize fields, respectively. Therefore, ~1.2 and 1.52% of P fertilizer was lost *via* leaching and subsurface runoff processes, respectively. Although limitation existed in quantitatively evaluating the loss of N in gaseous forms and the losses of N and P *via* leaching and subsurface runoff processes, it is still informative that surface runoff is one of the key liquid pathways for N loss from the paddy and maize fields. Moreover, most of the P imported from fertilizers was fixed in the soil particulate instead of releasing out *via* the runoff or leaching process.

### Effects of Fertilization, Irrigation, and Tillage-Based Management Strategies on N and P Runoff Loss Reduction and Its Recommendations on Control Measures

In this study, N and P losses were found to be consistently correlated with fertilization management strategies. This finding indicates that N and P losses were mainly derived from the nutrients in fertilizers instead of background soil N and P content, although fertilization, in turn, improved the growth status of the crops and grain yields (Tables 1, 2). This study further revealed that considerable proportions of agricultural N and P were lost during the stages of basal fertilization and first top dressing. Meanwhile, frequent rainfall events following fertilizer application triggered significant N and P losses from cropping systems. Therefore, it is suggested that decreasing the fertilization rate during the basal and first topdressing stages and avoiding fertilizer application around the time of a heavy rainfall event are effective methods for controlling N and P losses. Zhao et al. (2020) reported that an integration of adjusting optimal crop demand for fertilizer and fertilization time led to a total reduction in N loss by 31.6% from paddy fields.

**Figure 6:**
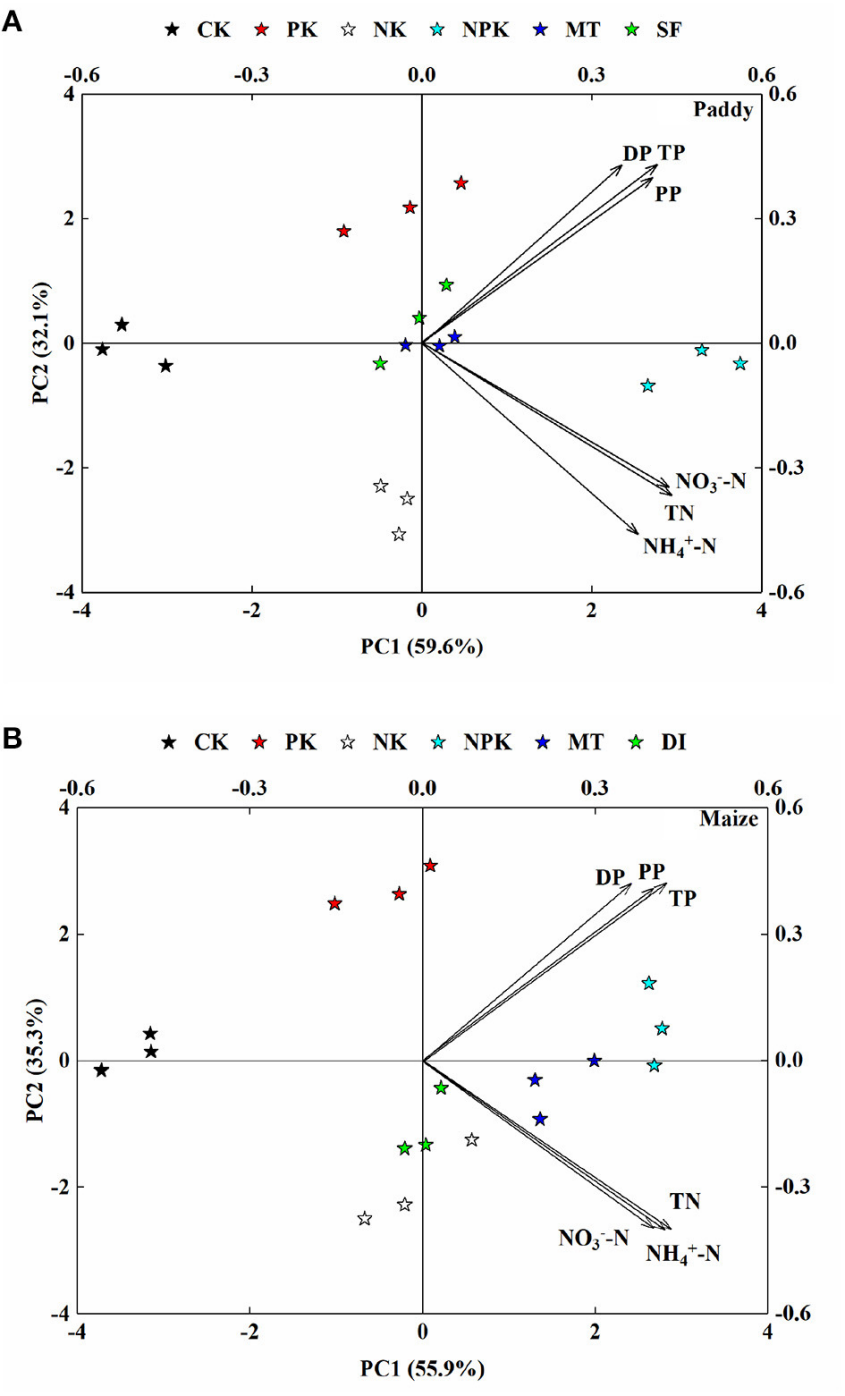
Principal component analysis (PCA) of TN, ${\text{NH}}_{4}^{+}$-N, ${\text{NO}}_{3}^{-}$-N, TP, PP, and DP runoff losses from **(A)** paddy and **(B)** maize under different agricultural treatment conditions. Arrows represent different formations of nitrogen (N) and phosphorus (P), and symbols with the same color in the PCA plot indicate the three replicates of each matrix.

According to the monitoring, N fertilizer plays an essential role in determining grain yields and other agronomic traits, such as 1,000-grain weight and seed-setting rate of paddy and the barren ear tip, kernel row number, 100-grain weight, and seed-setting rate of maize (see Tables 1, 2 and Figure B3 in Appendix B). By contrast, the application of P fertilizer had insignificant effect on grain yields and other agronomic traits. As noted above, considerable proportions of P (78.15–85.88%) were found to be fixed in the soil rather than absorbed by crops, resulting in a low P use efficiency of 12.35% (Figure 5). Ma et al. (2018) also reported significant accumulation of P in agricultural soils in China because of continuous P surplus in recent decades. Therefore, decreasing the rate of P fertilization is a feasible method of minimizing negative environment impacts without adversely affecting crop yields. As previously noted, ${\text{NO}}_{3}^{-}$-N and ${\text{NH}}_{4}^{+}$-N were the main forms of N losses from paddy and maize fields, respectively, whereas PP was the predominant form in surface runoff losses from both the paddy and maize fields. The PCA analysis confirmed these results, as shown in Figure 6. Therefore, the application of “edge-of-field” management practices, such as ecological ditch or constructed wetland, would help prevent soil erosion or mitigate losses in N and P in specific forms.

Apart from considering fertilization optimization, controlled managements related to irrigation and tillage modes were also applied in this study to provide references for developing appropriate ANPS pollution control strategies. According to the PCA analysis, controlled groups of water-saving irrigation (i.e., SF and DI) and conservation tillage (i.e., MT) were distributed distantly from the NPK groups along the PC1 axis (Figure 6). This result suggests that shallow-wet and drip irrigation modes and minimum tillage-based strategies led to a significant reduction in N and P losses at the same fertilization levels as compared with the groups with conventional managements. Compared with N and P losses in the conventional irrigation groups (i.e., NPK), N and P losses were reduced by around 31.69 and 40.01%, respectively, in the paddy field using shallow-wet irrigation, and 17.09 and 44.35%, respectively, in the maize field using drip irrigation (Figure 3). A previous study on paddy-based cropping system demonstrated a 45.8 and 21.9% reduction in N and P losses with shallow-wet irrigation strategy by decreasing irrigation frequency by 42.3% and amount of irrigation by 41.7% (Qi et al., 2020). The ranking of N and P losses based on PCA factor scores for different agriculture treatments are shown in Table A6 in Appendix A. Water-saving irrigation strategies (SF in the paddy field and DI in the maize field) obtained lower scores than conservation tillage strategies, suggesting that water-saving irrigation strategies are more effective than conservation one in reducing surface runoff losses of N and P from the paddy and maize fields. As for the conservation tillage application, the losses of N and P *via* surface runoff were found to be reduced by 24.4 and 33.75%, respectively, in the paddy field using the minimum tillage transplanting strategy (Figure 3). No obvious reduction in N loss was detected in the maize field using the minimum tillage transplanting strategy, but P loss was reduced by around 23.39%.

The outcomes from this study are expected to advance the understanding of the characteristics of agricultural N and P losses *via* surface runoff from paddy and maize cropping systems at Dongjiang Basin in South China. Notably, the effects of fertilization, irrigation, and tillage-based management on N and P losses were studied together with both crops (e.g., agronomic traits and yields) and characteristics of N and P losses (e.g., N/P loss forms and main pathways) in this study. Such knowledge will be valuable for formulating more targeted strategies to reduce N and P runoff losses, thereby effectively controlling ANPS pollution in the South China region with rain-prone subtropical monsoon climate and acidic red soil. Further studies may be needed to assess the loss in gaseous forms of N as well as losses in N and P *via* leaching and subsurface runoff processes.

## Conclusions

The main conclusions of this study are outlined below:

- Agricultural N and P losses from the maize field *via* surface runoff amounted to 27.85 and 1.24 kg ha^−1^ year^−1^, respectively, while those from the paddy field amounted to 15.37 and 0.8 kg ha^−1^ year^−1^, respectively. Frequent rainfall events following fertilization at the basal and first topdressing stages drove N and P losses from cropping systems *via* surface runoff.
- The main forms of N that were lost were ${\text{NO}}_{3}^{-}$-N and ${\text{NH}}_{4}^{+}$-N from the paddy and maize fields, respectively, whereas PP was the dominant form of P losses *via* surface runoff losses from both the paddy and maize fields. Fertilizers containing N instead of P plays a more important role in grain yields and other agronomic traits. Decreasing the rate of P fertilization is a feasible method for minimizing negative environment impacts without adversely affecting crop yields.
- The environmental fate of agricultural N and P imported from fertilizer was quantitatively analyzed, which was rarely achieved in previous literature. Approximately 26.22 and 37.48% of N fertilizer was recovered from the grains and straw of paddy and maize, respectively, whereas only 12.35 and 19.51% of P fertilizer was recovered during the crop harvesting stage. Surface runoff was found to be a key liquid pathway of N loss from the paddy and maize fields. Furthermore, most of the P imported from fertilizers was fixed in the soil particulate (78.15–85.88%) instead of lost *via* runoff (0.57–0.82%) or leaching processes (1.2–1.52%). This is probably because of the high Fe and Al content in typical red soil, which is prevalent at Dongjiang Basin.
- A PCA analysis confirmed that N and P losses were mainly derived from the nutrients in fertilizers rather than background soil N and P content. Controlled management relating to fertilization, irrigation, and tillage modes are feasible measures for reducing agricultural N and P losses, thereby effectively controlling ANPS pollution.

## Data Availability Statement

The original contributions presented in the study are included in the article/Supplementary Material, further inquiries can be directed to the corresponding author/s.

## Author Contributions

ZZh, YL, and FZ contributed to conception and design of the study. ZZu and ZhZ organized the database. JM, ZH, and TC performed the statistical analysis. HX performed the PCA analysis. FZ wrote the first draft of the manuscript. CC, XY, JW, and YW wrote sections of the manuscript. All authors contributed to manuscript revision, read, and approved the submitted version.

## Conflict of Interest

The authors declare that the research was conducted in the absence of any commercial or financial relationships that could be construed as a potential conflict of interest.

## Publisher's Note

All claims expressed in this article are solely those of the authors and do not necessarily represent those of their affiliated organizations, or those of the publisher, the editors and the reviewers. Any product that may be evaluated in this article, or claim that may be made by its manufacturer, is not guaranteed or endorsed by the publisher.
