# Supplementary material for: Runoff Losses in Nitrogen and Phosphorus From Paddy and Maize Cropping Systems: A Field Study in Dongjiang Basin, South China
Source: Front Plant Sci. 2021 Aug 10;12:675121. doi: 10.3389/fpls.2021.675121 (PMC8384078; doi:10.3389/fpls.2021.675121)
Supplement: Supplementary file 1 [file Data_Sheet_1.docx]

**Supplementary Data**

**Runoff losses of nitrogen and phosphorus from** **paddy and maize cropping systems: A field study in Dongjiang Basin, South China**

**Feifan ZENG**^1^**, Zheng ZUO**^1^**, Juncheng MO**^1^**, Chengyu CHEN**^1^**, Xingjian YANG**^1^**,** **Jinjin WANG**^1^**, Huijuan XU**^1^**, Yi WANG**^2^**, Zhongqiu ZHAO**^1^**, Zheng HU**^1^**, Tianyi CHEN**^1^**, Yongtao LI**^1, 3,*^, **Zhen ZHANG**^1,*^

^1^ College of Natural Resources and Environment, Joint Institute for Environmental Research & Education, South China Agricultural University, Guangzhou, China.

^2^ Changsha Research Station for Agricultural & Environmental Monitoring and Key Laboratory of Agro-ecological Processes in Subtropical Regions, Institute of Subtropical Agriculture, Chinese Academy of Sciences, Hunan, China.

^3^ Agro-Environmental Protection Institute, Ministry of Agriculture, Tianjin, China.

*Corresponding authors:

Yongtao LI (Email: yongtao@scau.edu.cn);

Zhen ZHANG (Email: [zzhangal@scau.edu.cn](mailto:zzhangal@scau.edu.cn), zhangzhen_23102@163.com).

**Appendix A**

**Table A.1** Soil (0-30 cm) physicochemical properties at the study site (n = 3)

| Clay (%) | Silt (%) | Sand (%) | Agrotype | Bulk density  (g cm^-3^) | pH | Organic matter  (g kg^-1^) | Total N  (g kg^-1^) | Total P  (g kg^-1^) | Available N (mg kg^-1^) | Olsen P (mg kg^-1^) |
| --- | --- | --- | --- | --- | --- | --- | --- | --- | --- | --- |
| 14.6 | 15.9 | 69.5 | Sandy loam | 1.26±0.05 | 5.83±0.21 | 15.49±1.59 | 1.05±0.74 | 0.22±0.03 | 60.97±4.39 | 19.47±1.85 |

**Table A.2** Different agricultural treatment design for paddy and maize fields

| Crop type | Treatment | Fertilizer application | | | Tillage pattern | Irrigation pattern |
| --- | --- | --- | --- | --- | --- | --- |
|  |  | N  (kg ha^-1^) | P  (kg ha^-1^) | K  (kg ha^-1^) |  |  |
| Paddy | CK ^a^ | 0 | 0 | 0 | Conventional | Conventional |
|  | PK ^b^ | 0 | 67 | 114 | Conventional | Conventional |
|  | NK ^c^ | 148 | 0 | 114 | Conventional | Conventional |
|  | NPK ^d^ | 148 | 67 | 114 | Conventional | Conventional |
|  | MT ^e^ | 148 | 67 | 114 | Minimum | Conventional |
|  | SF ^f^ | 148 | 67 | 114 | Conventional | Shallow-Wet |
| Maize | CK ^a^ | 0 | 0 | 0 | Conventional | Conventional |
|  | PK ^b^ | 0 | 34 | 140 | Conventional | Conventional |
|  | NK ^c^ | 132 | 0 | 140 | Conventional | Conventional |
|  | NPK ^d^ | 132 | 34 | 140 | Conventional | Conventional |
|  | MT ^e^ | 132 | 34 | 140 | Minimum | Conventional |
|  | DI ^g^ | 132 | 34 | 140 | Conventional | Drip |

^a^ CK refers to control group. ^b^ PK refers to phosphorus and potassium fertilizer application.

^c^ NK refers to nitrogen and potassium fertilizer application.

^d^ NPK refers to nitrogen, phosphorus and potassium fertilizer application.

^e^ MT refers to minimum-tillage transplanting.

^f^ SF refers to shallow-wet irrigation. ^g^ DI refers to drip irrigation.

**Table A.3** Runoff water volume of paddy field

| Data | Runoff water volume (L) | | | | | |
| --- | --- | --- | --- | --- | --- | --- |
|  | CK ^a^ | PK ^b^ | NK ^c^ | NPK ^d^ | MT ^e^ | SF ^f^ |
| 19-8-2019 | 853.1±7.5 | 880.0±11.8 | 866.8±11.1 | 907.8±5 | 899.4±1.6 | 865.7±8.8 |
| 27-8-2019 | 877.7±5.4 | 884.8±8.5 | 878.4±10.1 | 880.9±9.3 | 876.2±17.5 | 874.7±14.1 |
| 3-9-2019 | 863.7±9.5 | 869.3±3.6 | 857.7±8.5 | 859.5±14.6 | 870.4±20.9 | 877.8±14 |
| 18-9-2019 | 681.2±14.2 | 707.6±9.6 | 673.9±6.6 | 684.9±11.7 | 689.5±8.6 | 681.2±13.3 |
| 10-10-2019 | 718.1±9.6 | 739.3±7.7 | 727.9±17.9 | 727.2±9.9 | 719.6±12.2 | 725.2±17.6 |
| 18-11-2019 | 419.5±19.3 | 436.9±10.6 | 430.4±14.7 | 425.3±15.5 | 417.9±17.9 | 415.3±3.3 |
| 7-4-2020 | 853.0±32.9 | 839.3±10.7 | 864.0±8.2 | 861.0±37.0 | 856.7±28.3 | 869.7±11.6 |
| 13-4-2020 | 543.0±16.3 | 583.3±9.8 | 568.0±14.2 | 540.0±24.2 | 511.7±10.1 | 521.3±22.1 |
| 23-4-2020 | 664.7±24 | 648.5±30.5 | 646.0±38.6 | 625.3±11.9 | 631.7±8.2 | 637.7±22.7 |
| 27-5-2020 | 860.3±20.1 | 832.0±21.5 | 843.3±26.7 | 833.3±31.8 | 868.7±18.4 | 849.3±29.3 |
| 2-6-2020 | 611.3±11.8 | 633.3±33.2 | 649.7±29.8 | 678.7±18.9 | 641.0±38.9 | 650.3±27.7 |
| 9-6-2020 | 431.3±22.5 | 467.7±24.6 | 469.3±7.6 | 481.7±14.7 | 462.7±38.4 | 444.3±11.4 |
| 18-6-2020 | 724±10.2 | 743.3±33.9 | 753.7±17.3 | 748.7±35.1 | 740.7±35.1 | 747.3±14.1 |
| 29-6-2020 | 548±28.2 | 567.7±43.6 | 567.7±13.9 | 531.3±9.2 | 554.3±37.7 | 576.7±17.6 |
| 14-7-2020 | 462.0±36.7 | 437.0±30.0 | 447.0±24.7 | 493.7±2.6 | 427.0±19.0 | 449.7±11.9 |

^a^ CK refers to control group. ^b^ PK refers to phosphorus and potassium fertilizer application. ^c^ NK refers to nitrogen and potassium fertilizer application.

^d^ NPK refers to nitrogen, phosphorus and potassium fertilizer application. ^e^ MT refers to minimum-tillage transplanting. ^f^ SF refers to shallow-wet irrigation

**Table A.4** Runoff water volume of maize field

| Data | Runoff water volume (L) | | | | | |
| --- | --- | --- | --- | --- | --- | --- |
|  | CK ^a^ | PK ^b^ | NK ^c^ | NPK ^d^ | MT ^e^ | DI ^f^ |
| 5-7-2019 | 943.4±6.6 | 943.7±3.8 | 944.9±4.6 | 946.2±6 | 944.3±8.5 | 956.1±0.9 |
| 11-7-2019 | 932.2±18.3 | 938.3±19.0 | 925.5±10.8 | 919.1±4.8 | 949.9±8.9 | 943.5±15.2 |
| 23-7-2019 | 935.1±13.4 | 933.0±10.0 | 930.8±7.4 | 924.3±1.8 | 934.1±11.1 | 935.4±5.4 |
| 7-8-2019 | 936.8±3.1 | 917.2±5.9 | 917.6±9.0 | 923.9±14.3 | 921.1±4.6 | 918.7±3.9 |
| 19-8-2019 | 881.8±12.3 | 874.0±11.0 | 879.7±2.7 | 873.0±7.9 | 876.4±5.5 | 873.2±12.4 |
| 27-8-2019 | 755.9±15.4 | 746.1±14.9 | 760.4±12.6 | 752.8±13.6 | 763.0±20.6 | 751.4±17.3 |
| 3-9-2019 | 704.6±8.2 | 689.6±16.3 | 703.2±19.4 | 686.3±15.7 | 698.7±4.4 | 704.8±6.3 |
| 10-10-2019 | 492.9±17.0 | 482.3±18.3 | 494±18.2 | 478.2±9.3 | 489.3±16.7 | 490.1±14.3 |
| 18-11-2019 | 485.7±24.6 | 476.0±19.1 | 494.4±21.2 | 504.0±13.3 | 480.1±12.2 | 176.2±11.3 |
| 10-12-2019 | 351.5±14.6 | 358.3±6.4 | 349.7±7.4 | 342.2±4.9 | 356.5±6.0 | 149.1±14.1 |
| 18-12-2019 | 258.5±6.8 | 261.2±3.9 | 246.2±4.2 | 240.6±8.2 | 234.5±2.0 | 61.0±6.7 |
| 25-12-2019 | 203.4±13.4 | 204.1±13.4 | 201.4±12.8 | 197.3±16.6 | 199.7±5.3 | 63.3±3.2 |
| 27-5-2020 | 841.1±29.1 | 864.7±17.9 | 825.2±14.4 | 853.4±36.5 | 868.3±20.3 | 826.7±8.6 |
| 2-6-2020 | 652.2±36.6 | 670.9±21.4 | 655.3±20.9 | 657.7±33.2 | 634.1±36.0 | 670.1±22.2 |
| 9-6-2020 | 442.6±21.1 | 442.9±5.7 | 437.2±6.6 | 440.3±30.6 | 467.3±10.9 | 481.4±12.6 |
| 18-6-2020 | 732.6±10.9 | 775.6±5.5 | 744.7±9.4 | 723.9±19.7 | 750.5±33.7 | 783.4±14.1 |
| 29-6-2020 | 575.2±14.3 | 539.5±29.2 | 558.5±29.4 | 532.4±1.9 | 549.2±22.6 | 530.8±14.4 |
| 14-7-2020 | 448.3±35.7 | 455.0±32.2 | 441.1±22.3 | 461.1±18.8 | 459.2±8.4 | 438.8±37.4 |

^a^ CK refers to control group. ^b^ PK refers to phosphorus and potassium fertilizer application. ^c^ NK refers to nitrogen and potassium fertilizer application.

^d^ NPK refers to nitrogen, phosphorus and potassium fertilizer application. ^e^ MT refers to minimum-tillage transplanting. ^f^ DI refers to shallow-wet irrigation

**Table A.5** Supplementary information of paddy and maize growing seasons

| Crop type | Species | Growing seasons | N fertilizer application ^a^ | P fertilizer application |
| --- | --- | --- | --- | --- |
| Paddy | Huahang 51 | August 15, 2019 to November 18, 2019 | Basal fertilization:  August 14, 2019  First topdressing:  September 25, 2019  Second topdressing:  October 25, 2019 | August 14, 2019 |
|  |  | April 3, 2020 to July 14, 2020 | Basal fertilization:  April 3, 2020  First topdressing:  May 26, 2020  Second topdressing:  June 14, 2020 | April 3, 2020 |
| Maize | Yuetian 16 | July 2, 2019 to September 3,2019 | Basal fertilization:  July 1, 2019  First topdressing:  August 2, 2019  Second topdressing:  August 24, 2019 | July 1, 2019 |
|  |  | October 2, 2019 to  December 25, 2019 | Basal fertilization:  October 1, 2019  First topdressing:  November 4, 2019  Second topdressing:  December 14, 2019 | October 1, 2019 |
|  |  | May 24,2020 to  July 14, 2020 | Basal fertilization:  May 24, 2020  First topdressing:  June 4, 2020  Second topdressing:  June24, 2020 | May 24, 2020 |

^a^ In each growing season, the application of N fertilizer included basal fertilization application and two topdressing application, whereas, P fertilizer was applied in one time at basal fertilization stage.

**Table A.6** Nitrogen and phosphorus losses ranking based on PCA Factor scores different agriculture treatment

| Ranking | Paddy | Maize |
| --- | --- | --- |
| 1 | NPK ^a^ (1.81) | NPK ^a^ (1.70) |
| 2 | PK ^b^ (0.58) | PK ^b^ (0.74) |
| 3 | MT ^c^ (0.08) | MT ^c^ (0.72) |
| 4 | SF ^d^ (0.06) | DI ^g^ (-0.36) |
| 5 | NK ^e^ (-1.02) | NK ^e^ (-0.76) |
| 6 | CK ^f^ (-2.07) | CK ^f^ (-1.82) |

^a^ NPK refers to nitrogen, phosphorus and potassium fertilizer application.

^b^ PK refers to phosphorus and potassium fertilizer application.

^c^ MT refers to minimum-tillage transplanting.

^d^ SF refers to shallow-wet irrigation.

^e^ NK refers to nitrogen and potassium fertilizer application.

^f^ CK refers to control group.

^g^ DI refers to drip irrigation.

**Appendix B**

**

**

**Fig. B.1** Monthly and maximum daily precipitation from July, 2019 to July, 2020.


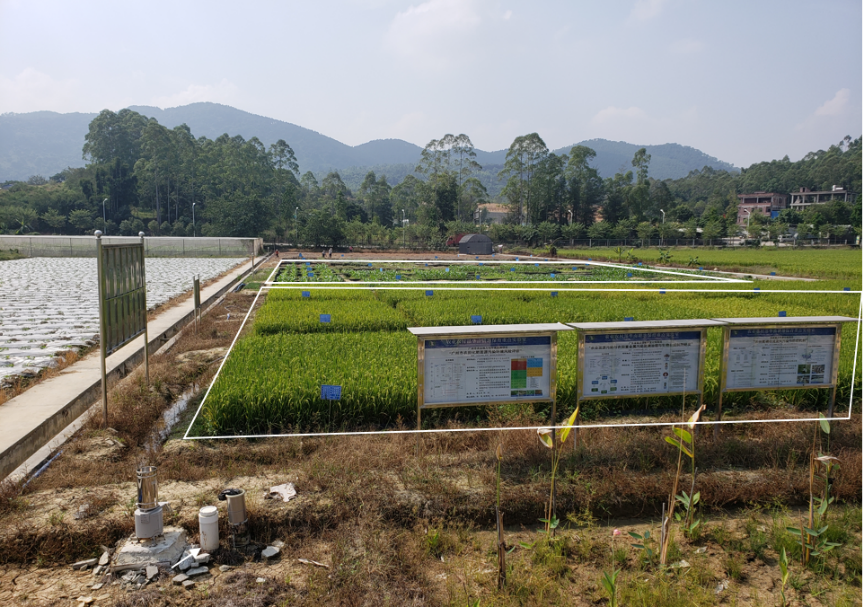


Maize plot

Paddy plot

Small climate station

Groundwater-level observation well

(a)


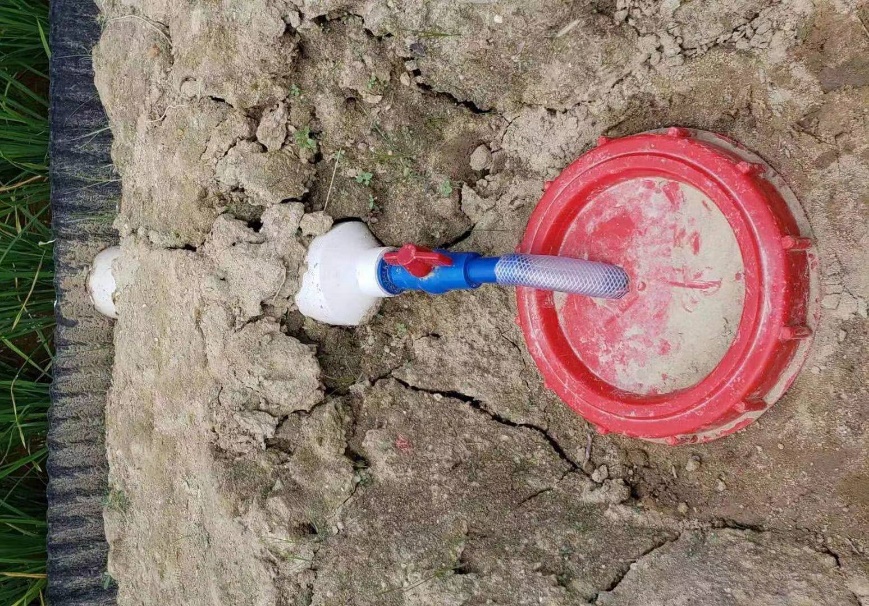


Overflow bucket

(b)

(c)

Miniature soil sampler

**Fig. B.2** (a) Panoramagram of study area, (b) overflow bucket, and (c) miniature soil moisture sampler.


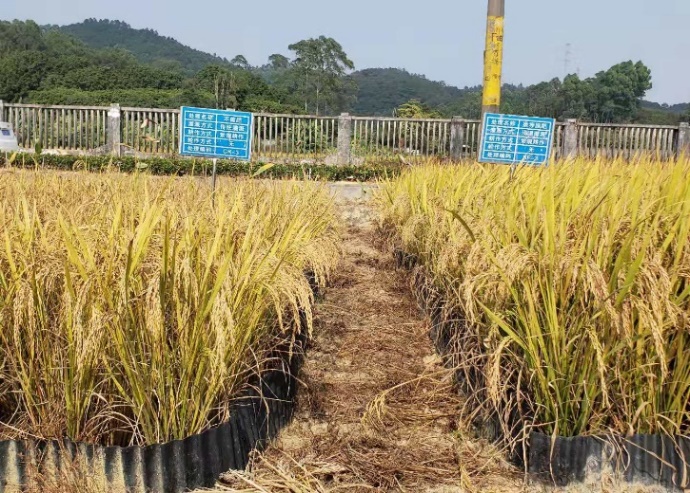


NK

NPK

(a)


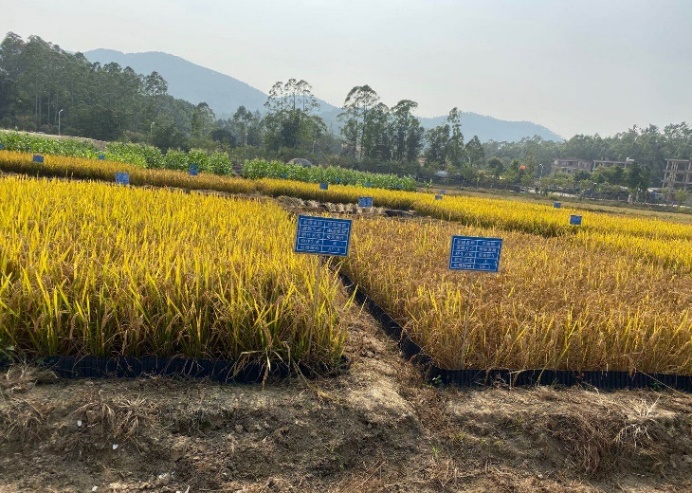


NPK

PK

(b)


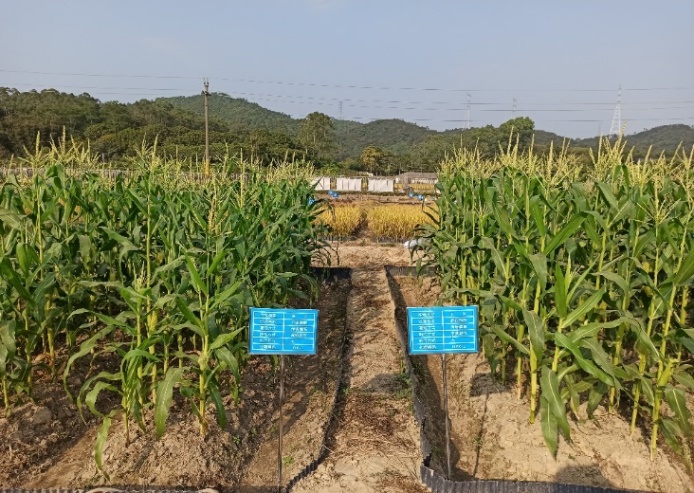


NPK

NK

(c)


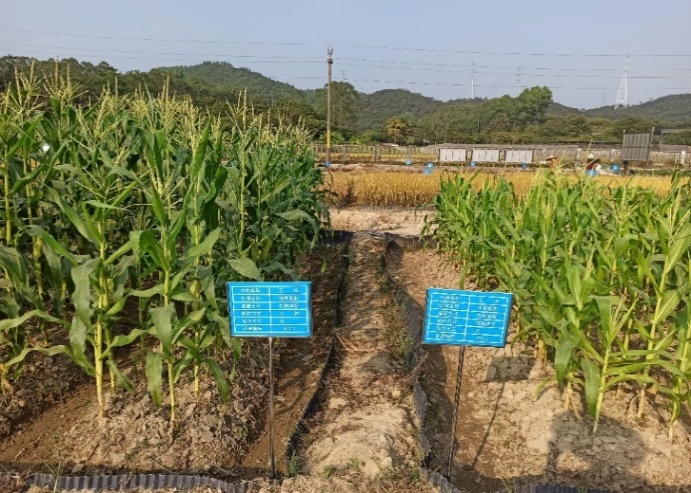


(d)

NPK

PK

**Fig. B.3** Paddy rice grown differences between (a) NPK and NK group and between (b) NPK and PK group; maize grown difference between (c) NPK and NK group and between (d) NPK and PK group.

**





**

**





**

**Fig. B.4** Soil (a) TN, (b) available N, (c) TP, (d) Olsen P, (e) pH, and (f) organic matter in the 0-30 and 30-60 cm depths of paddy under different agricultural treatments. Means within the same soil depth followed by the different letters are significantly different (LSD, *p < 0.05*).

**





**

**





**

**Fig. B.5** Soil (a) TN, (b) available N, (c) TP, (d) Olsen P, (e) pH, and (f) organic matter in the 0-30 and 30-60 cm depths of maize under different agricultural treatments. Means within the same soil depth followed by the different letters are significantly different (LSD, *p < 0.05*).







**



**

**Fig. B.6** TN concentration in leaching water of (a) paddy and (b) maize under different agricultural treatments (0-30 and 30-60 cm depths). TP concentration in leaching water of (c) paddy and (d) maize under different agricultural treatments (0-30 and 30-60 cm depths).












**Fig. B.7** Correlation between precipitation and runoff loss of TN in (a) paddy and (b) maize, correlation between precipitation and runoff loss of TP in (c) paddy and (d) maize
